# Temporal dynamics of migration‐linked genetic variation are driven by streamflows and riverscape permeability

**DOI:** 10.1111/mec.15367

**Published:** 2020-02-17

**Authors:** Suzanne J. Kelson, Michael R. Miller, Tasha Q. Thompson, Sean M. O’Rourke, Stephanie M. Carlson

**Affiliations:** ^1^ Environmental Science, Policy, and Management University of California Berkeley CA USA; ^2^ Department of Animal Science University of California Davis CA USA

**Keywords:** landscape genetics, life history, *Oncorhynchus mykiss*, partial barrier, partial migration, river network

## Abstract

Landscape permeability is often explored spatially, but may also vary temporally. Landscape permeability, including partial barriers, influences migratory animals that move across the landscape. Partial barriers are common in rivers where barrier passage varies with streamflow. We explore the influence of partial barriers on the spatial and temporal distribution of migration‐linked genotypes of *Oncorhynchus mykiss*, a salmonid fish with co‐occurring resident and migratory forms, in tributaries to the South Fork Eel River, California, USA, Elder and Fox Creeks. We genotyped >4,000 individuals using RAD‐capture and classified individuals as resident, heterozygous or migratory genotypes using life history‐associated loci. Across four years of study (2014–2017), the permeability of partial barriers varied across dry and wet years. In Elder Creek, the largest waterfall was passable for adults migrating up‐river 4–39 days each year. In this stream, the overall spatial pattern, with fewer migratory genotypes above the waterfall, remained true across dry and wet years (67%–76% of migratory alleles were downstream of the waterfall). We also observed a strong relationship between distance upstream and proportion of migratory alleles. In Fox Creek, the primary barrier is at the mouth, and we found that the migratory allele frequency varied with the annual timing of high flow events. In years when rain events occurred during the peak breeding season, migratory allele frequency was high (60%–68%), but otherwise it was low (30% in two years). We highlight that partial barriers and landscape permeability can be temporally dynamic, and this effect can be observed through changing genotype frequencies in migratory animals.

## 1 INTRODUCTION

Landscape features shape patterns of species composition and genetic diversity. Fragmented landscapes, natural or artificial, are characterized by barriers to movement and dispersal. Many barriers are almost or completely impassible, such as roads (Holderegger & Di Giulio, 2010; Shepard, Kuhns, Dreslik, & Phillips, 2008) or dams (Fullerton et al., 2011; Sheer & Steel, 2006), which impede movement and reduce or eliminate gene flow, facilitating genetic divergence between populations (Manel & Holderegger, 2013). Other “partial” barriers are more permeable for moving organisms, such as low‐density human development that discourages migration of ungulates (Sawyer et al., 2013), or sunny, open‐canopy patches that cause water‐loss and discourage movement of woodland salamanders (Peterman, Connette, Semlitsch, & Eggert, 2014). Exposure to multiple partial barriers may be cumulatively as important as one complete barrier in shaping patterns of movement and genetic diversity (Apgar, Pearse, & Palkovacs, 2017). In addition, the permeability of partial barriers is likely to have a strong temporal component, with permeability varying on scales from hours to decades. For example, temporary flooding can promote movement for the Australian freshwater turtle between otherwise disconnected, temporary wetlands (Roe, Brinton, & Georges, 2009). Migratory animals in particular may be affected by partial barriers, as they rely on passage across landscapes to complete their life cycle (Fahrig, 2007; Tucker et al., 2018).

Rivers offer interesting systems for exploring temporal variation in barrier permeability for several reasons. First, partial barriers such as small waterfalls, logjams and culverts are widespread and may be even more prevalent than complete barriers in river networks (Kemp & O’Hanley, 2010; Meixler, Bain, & Todd Walter, 2009). Furthermore, upstream‐ and downstream‐moving aquatic organisms will encounter and be influenced by all in‐stream barriers, because there is no way to circumvent barriers as in terrestrial or marine systems. Such partial barriers can limit the upstream distribution of aquatic invertebrates (Blanco & Scatena, 2006; Kerby, Riley, Kats, & Wilson, 2005) and fishes (Fausch, Rieman, Dunham, Young, & Peterson, 2009), which can then result in divergent communities above and below barriers (Anderson, Freeman, & Pringle, 2006; Perkin & Gido, 2012). Partial barriers can also lead to genetic divergence in aquatic species, reflecting long‐term patterns of gene flow, often resulting in reduced genetic diversity above barriers (Carlsson & Nilsson, 2011; Wofford, Gresswell, & Banks, 2005; Yamamoto, Morita, Koizumi, & Maekawa, 2004). Furthermore, barrier permeability is likely to change on short timescales because river flows rise and fall with precipitation inputs. Seasonal and interannual variation in river flow may inhibit or facilitate animal movement across waterfalls (Powers & Orsborn, 1985; Reiser, Huang, Beck, Gagner, & Jeanes, 2006), road culverts (Belford & Gould, 1989) and weirs (Russon & Kemp, 2011). The movement of migratory organisms may be especially impacted by temporal variation in flow conditions at partial barriers, with low flows often limiting the ability of migratory animals to reach upstream breeding or rearing habitats, as in diadromous aquatic invertebrates (Resh, 2005) and fishes (Rolls, 2011). Furthermore, river systems are vulnerable to climate change, as changes in the timing, magnitude and type of precipitation (rain or snow) will shift the timing and magnitude of elevated stream flow events and floods (Dettinger, 2011; Mallakpour & Villarini, 2015; Stewart, Cayan, & Dettinger, 2004). Quantifying how among‐year variation in weather influences barrier permeability and the subsequent ability of migratory species to move freely throughout river networks will be critical for predicting the impacts of climate change on migration patterns and species distributions.

One migratory fish common to rivers across the northern Pacific Rim is the salmonid *Oncorhynchus mykiss*. This species is partially migratory, meaning that some individuals migrate to the ocean (i.e., anadromous “steelhead” trout) whereas others complete their entire life history in freshwater (i.e., resident “rainbow” trout). In general, migratory *O. mykiss* occupy lower elevation streams with easy access to the ocean, while resident *O. mykiss* dominate further upstream (Berejikian, Campbell, & Moore, 2013; Kendall, McMillan, & Sloat, 2014; Narum, Zendt, Graves, & Sharp, 2008) and above impassible barriers (Pearse et al., 2009; Thrower & Joyce, 2004). Like other migratory salmonid fishes, steelhead trout migrate from the ocean to freshwater to breed, and swim upstream to seek out breeding sites, ideally where their offspring will experience reduced competition and densities (Fleming & Reynolds, 2003). While large barriers mark step‐wise transitions between migratory and resident life history forms (Pearse et al., 2009), it is less clear how small partial barriers influence the distribution of the two forms in streams where they co‐occur. The recent discovery of a genomic region associated with life history type (i.e., migratory vs. resident) in *O. mykiss* (Pearse et al., 2019; Pearse, Miller, Abadía‐Cardoso, & Garza, 2014) has opened the door to exploring the influence of partial barriers on genetic diversity associated with life history in *O. mykiss* at fine spatial and temporal scales.

Landscape features can shape both neutral genetic structure and the distribution of adaptive variation within a species (Davis, Epps, Flitcroft, & Banks, 2018; Grummer et al., 2019; Orsini, Andrew, & Eizaguirre, 2013). While landscape genetic studies in rivers increasingly consider adaptive variation (Brauer, Unmack, Smith, Bernatchez, & Beheregaray, 2018; Micheletti, Matala, Matala, & Narum, 2018; Vincent, Dionne, Kent, Lien, & Bernatchez, 2013), few studies have directly compared patterns of neutral genetic variation with patterns of variation at loci associated with adaptive phenotypic variation (but see Hand et al., 2016; Keller, Taverna, & Seehausen, 2011; O’Malley, Jacobson, Kurth, Dill, & Banks, 2013). This comparison could improve our understanding of the mechanisms that either facilitate or restrict gene flow in the face of selection and adaptive divergence on life history characteristics (e.g., migratory vs. resident life histories) in fragmented river networks and landscapes.

Here we explored how partial barriers influence migration‐associated genetic diversity in *O. mykiss* through space and time in two streams. First, we characterized genetic structure at putatively neutral loci to explore patterns of gene flow and genetic divergence. Second, we characterized the spatial distribution of genotypes at the life history‐associated loci in the two streams. In particular, we tested if the frequency of migration‐associated alleles decreased with distance upstream. Next, we explored the influence of partial barriers, including natural waterfalls and tributary confluences, on the distribution of migratory and resident alleles. We predicted that partial barriers would reduce the frequency of migratory alleles found upstream. Finally, we explored interannual variation in the permeability of these partial barriers. We predicted that in years with less precipitation, there would be fewer passage opportunities, and migratory alleles would be concentrated downstream, below partial barriers, in comparison to wet years, when landscape permeability is higher and passage opportunities are more frequent. Together these questions allowed us to explore how trait‐linked genetic diversity is influenced by partial barriers in stream systems across years with different patterns of precipitation and stream flow.

## 2 MATERIALS AND METHODS

### 2.1 System and study streams

**Figure 1:**
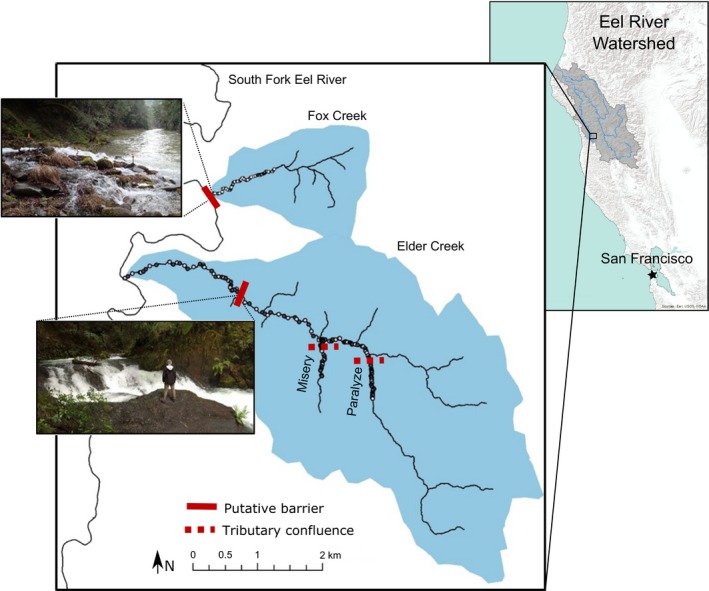
Elder Creek and Fox Creek are tributaries to the South Fork Eel River watershed in northern California, USA. Circles represent sample pools, which were spatially stratified to encompass the entire length of the stream occupied by *Oncorhynchus mykiss*. White pools were included in genetic analyses for all years (2014–2017), while dark grey pools were included in 2014 only [Colour figure can be viewed at http://wileyonlinelibrary.com]

We studied the distribution of migratory and resident *Oncorhynchus mykiss* in two tributaries to the South Fork Eel River, Fox Creek and Elder Creek (Figure 1), both of which are located within the University of California Angelo Coast Range Reserve. Migratory *O. mykiss* (“steelhead trout”) rear for 1–3 years in freshwater before migrating to the ocean for feeding and rearing. After 1–3 years, migratory adults then return to freshwater to breed, swimming upstream, arriving in streams in the Eel River watershed from January to May (Brown, 1990; Trush, 1989). They build nests in the gravel and their juveniles emerge from April to June (S. Kelson, personal observation). Resident *O. mykiss* (“rainbow trout”) complete their entire life cycle in freshwater, often remaining in their natal stream. *O. mykiss* represent >99% of the fish biomass in the streams studied here.

Fox Creek is a small watershed, draining 2.7 km^2^,with step‐pool morphology (Montgomery & Buffington, 1997). Elder Creek is a larger watershed, draining 16.8 km^2^, with two fish‐bearing tributaries, Misery Creek (drainage area of 1.9 km^2^) and Paralyze Creek (4.9 km^2^; Figure 1). Elder Creek is characterized by pool‐riffle morphology in the lower reaches and step‐pool morphology in the upper reaches, including in both tributaries. The transition from pool‐riffle to step‐pool morphology occurs near the confluence with Misery Creek, 4.1 km upstream from the Elder Creek mouth.

We explored the influence of three partial barriers within Elder Creek and one partial barrier within Fox Creek on the upstream‐extent of migratory genotypes. The most downstream feature in Elder Creek is a large waterfall (3.1 m high from base to crest) that is a barrier to upstream movement of adult fish at most stream flows (Trush, 1989), and is located 2 km from the mouth (Figure 1, hereafter referred to as “Elder waterfall”). The second and third barriers are the two tributary junctions, the mouths of Misery and Paralyze creeks. There is a small elevational step to enter Misery Creek, and then a 1‐m‐high waterfall in the first 20 m upstream. There is a large, steep barrier at the mouth of Paralyze Creek (1.7 m high). There are no known barriers within Fox Creek, but the creek is elevated relative to the South Fork Eel River at their confluence, creating a partial barrier to upriver migrating steelhead at the mouth of the creek. While the creek mouth of Elder Creek is also elevated, the larger drainage area and higher flows of Elder Creek renders this step a passable barrier (Trush, 1989). The location of the potential landscape barriers in Fox and Elder creeks are illustrated in Figure 1.

### 2.2 Interannual variation in stream passage conditions

The study region experiences Mediterranean seasonality, which is characterized by high variability in precipitation among years and hence high variability in river flows (Cid et al., 2017). Consequently, we expected the permeability of partial barriers within these streams to vary among years. We classified our four study years (2014–2017) as “dry” or “wet” using the Drought Severity Classification Index (DSCI) data for the South Fork Eel River watershed from the National Drought Monitor (https://droughtmonitor.unl.edu). We calculated the average DSCI for each year during the steelhead migration and breeding season (January–May) (Brown, 1990; Trush, 1989), and considered years with a DSCI score of >250 (out of 500) as “dry” and years with a DSCI score of <250 as “wet.” We also used stream flow records from the USGS gauge on Elder Creek (gauge no. 11475560) to estimate interannual differences in stream flow and the opportunities for adult steelhead to ascend the aforementioned partial barriers. Trush (1989) observed that adult steelhead can ascend the largest waterfall in Elder Creek when flows are between 1.7 and 4.8 m^3^/s. This information allowed us to estimate the number of days that the Elder waterfall was passable to adult steelhead during the breeding season in each of our four study years. The mouth of Fox Creek and the Misery and Paralyze confluences within the Elder Creek watershed are probably passable at a broader range of stream flows.

### 2.3 Study pools

To collect tissue samples for genetic analyses, we sampled fish longitudinally from the entire fish‐bearing extent of each creek in four years, 2014–2017. In 2014, we mapped and numbered habitat units (pools) in each stream onto a 10‐m topographic map in the field. We sampled fish from ~20% of the pools in each stream, selecting study pools using a spatially stratified random sampling approach to ensure that sample pools extended from the mouth to the upper extent of fish in both streams. The surface area (m^2^) of each unit was measured within 2 weeks of fish sampling, and was estimated as pool length × average pool width, based on five evenly spaced width measurements. We calculated the stream distance from the pool to the mouth of the creek (Fox or Elder) in arcgis. The same pools were revisited each year, with a few exceptions due to natural alterations in the stream channel that made some pools inaccessible in later years. When this occurred, we replaced the original pool with the next upstream pool. This sampling protocol allowed us to compare changes in life history‐associated allele frequencies among years and locations.

### 2.4 Fish sampling

We sampled fish using three‐pass backpack electrofishing in each pool. Pools were blocked with nets before sampling and effort (seconds) was recorded for each pass. Using this method, we captured the majority of fish in most study pools. We used the fish abundance estimate combined with the pool surface area to estimate fish density (fish/m^2^). We also estimated abundance using the Leslie‐K three‐pass depletion method (Leslie & Davis, 1939; Ogle, 2016), and found that the total count of fish was highly correlated with three‐pass depletion estimate except in the subset of pools with very small catches, which led to unreliable depletion estimates (detailed in Kelson & Carlson, 2019). We therefore use total fish counts as our estimate of abundance. Fish sampling and habitat data are available on Dryad (Kelson, Miller, Thompson, O’Rourke, & Carlson, 2020).

At capture, we removed a small tissue sample (caudal fin clip), which was stored at room temperature on Whatman filter paper in a coin envelope for genetic analyses. At the same time, fish were measured for fork length (FL, in mm) and mass (to the nearest 0.01 g). We collected additional tissue samples from juvenile trout collected in the South Fork Eel River during sampling for other studies (Schaaf, Kelson, Nusslé, & Carlson, 2017), and a subset of those samples (*n* = 112, mean ± *SD* FL = 61 ± 36 mm) were included here as a reference to the tributary sites in a principal component analysis (PCA; see below).

### 2.5 DNA extraction and genotyping

We conducted genetic analyses on all of the tissue samples collected in 2014. For 2015–2017 samples, we included a subset of ~50% of the samples, where every‐other study pool was included in the final analysis. We chose to subset the samples in the later years after preliminary analyses from 2014 revealed consistent results with a smaller number of samples. In total, we analysed *n* = 4,517 fish. For analyses around changing genotype frequencies, we focused on *n* = 3,081 fish that were captured systematically during electrofishing surveys. A breakdown by year, location, sample pool and age class for these fish is given in Table 1. Raw sequence data are available at NCBI, SRA accession: PRJNA599015.

**Table 1:** Number of pools and fish that were included in genetic samples in 2014–2017 by sample location

Year	Location	Number of pools	Total number of fish	Number of age−0 fish
2014	Fox Creek	22	45	13
	Elder – Below waterfall	32	731	679
	Elder – Above waterfall	46	397	284
	Elder – Misery	29	87	61
	Elder – Paralyze	32	186	150
2015	Fox Creek	26	111	100
	Elder – Below waterfall	17	242	224
	Elder – Above waterfall	23	155	106
	Elder – Misery	11	26	15
	Elder – Paralyze	16	76	46
2016	Fox Creek	24	89	66
	Elder – Below waterfall	17	157	136
	Elder – Above waterfall	25	180	107
	Elder – Misery	14	23	11
	Elder – Paralyze	16	85	40
2017	Fox Creek	26	127	94
	Elder – Below waterfall	14	151	115
	Elder – Above waterfall	23	110	58
	Elder – Misery	13	29	12
	Elder – Paralyze	15	74	34

We conducted DNA extractions and restriction site‐associated DNA capture (RAD capture, or RAPTURE) using the methods and bait sets described in Ali et al. (2016). Briefly, DNA was extracted from caudal fin tissue using a bead‐based protocol, and *Sbf*I RAD libraries were prepared and captured through hybridization with 500 unique RAPTURE baits distributed across all 29 chromosomes in the *O. mykiss* genome. We used an Illumina HiSeq to sequence libraries using paired‐end 100‐bp (2014 samples) or 150‐bp reads (2015–2017 samples). We demultiplexed sequence data using custom scripts (Ali et al., 2016) and used the MEM algorithm of the burrows–wheeler aligner (bwa; Li & Durbin, 2009) with standard parameters to align sequences to a rainbow trout genome assembly (https://www.ncbi.nlm.nih.gov/assembly/GCF_002163495.1/). We used samtools (Li et al., 2009) to filter alignments (unmapped reads, supplementary alignments and nonprimary alignments were removed, and only properly‐paired reads were retained), sort alignments, remove PCR (polymerase chain reaction) duplicates (using both samtools [rmdup] and picard tools [MarkDuplicates], https://broadinstitute.github.io/picard/) and index binary alignment map files (see Table S2 for number of reads retained at each step).

We used Analysis of Next Generation Sequencing Data (angsd) for all RAPTURE sequencing data analyses (Korneliussen, Albrechtsen, & Nielsen, 2014). We inferred major and minor alleles of sites with a high probability of being variable (SNP *p* < 1e−6) from genotype likelihoods. We estimated allele frequencies assuming a fixed major but unknown minor allele (Kim et al., 2011). Sites were included if they had a minor allele frequency >0.05, and had data in a minimum of 50% of the samples. From here, we created two sets of genotype files for analyses, one that could be used for PCAs and include a maximum sample size without a bias in data quality per individual, and another that could be used for descriptive genetics. For the first genotype type, we used a single read sampling approach, where, for each individual, a single base from each site passing the above filters was randomly sampled and used for downstream analyses. This approach creates an “identify by state” (IBS) matrix and mediates the effects of coverage difference (number of sequence reads) between individuals and facilitates the use of samples with low coverage, thus allowing a larger number of samples to be included in downstream analyses than is possible with other approaches (see also Kelson, Miller, Thompson, O'Rourke, & Carlson, 2019). We used a discriminant analysis of principal components (DAPC) (Jombart, Devillard, & Balloux, 2010), on the IBS matrix with only SNPs on *Omy05* (*n* = 415 SNPs) to assign individuals to migratory, heterozygous or resident genotype groups (described further in Kelson et al., 2019). Second, we called genotypes for all SNPs located on the 500 RAPTURE baits (i.e., SNPs that were enriched during sequencing and therefore had relatively high coverage) using a uniform prior and posterior probability cutoff of 0.95 and refer to this approach as “called genotypes” (*n* = 473 SNPs). We used the called genotypes to calculate metrics of genetic diversity (described below). Genotype data sets used for analyses in this paper are available on Dryad (Kelson et al., 2020).

### 2.6 Genetic structure in partially migratory fish

We predicted that genetic structure would be weak at neutral loci for partially migratory populations. We calculated observed versus expected heterozygosity for each SNP in the called genotype data set (*n* = 473 SNPs) in the r package “adegenet” (Jombart, Kamvar, & Collins, 2011). We also calculated pairwise *F*_ST_ values (with *Omy05* SNPs removed) between Fox Creek and the regions of Elder Creek using called genotypes in “hierfstat” (Goudet & Jombart, ) in R. Next, to visualize population structure within these streams, we conducted a PCA on the IBS matrix for neutral SNPs (removing *Omy05* SNPs, *n* = 702 SNPs included) using the “adegenet” package in r. For this PCA, we used the IBS matrix that included SNPs that were missing data at a maximum of 20% of individuals.

### 2.7 Data analysis: influence of distance upstream and partial barriers on migration‐linked genetic diversity

We explored the spatial distribution of migratory alleles in each stream. First, we tested if migratory fish were less likely to be found upstream in our streams, a pattern which has been observed at larger geographical scales using genetic (Narum et al., 2008) and nongenetic methods (Berejikian et al., 2013). This pattern is predicted because upstream habitats tend to be more difficult for migratory fish to access, increasing the cost of migration. We addressed this question by relating the proportion of migratory alleles per study pool with distance upstream from the confluence with the South Fork Eel River, and predicted that there would be a negative relationship between the two. For each pool, we calculated the proportion of migratory alleles (individuals assigned a homozygous‐migratory genotype = 2 alleles, heterozygote = 1 allele, and homozygous‐resident = 0 alleles, divided by the total number of alleles, 2 per fish). We conducted a generalized linear regression, using a binomial distribution for proportions (response variable ranged from 0 to 1), with the proportion of migratory alleles as the response variable and distance upstream as the predictor variable. We calculated regressions separately for each year (*n* = 4) and creek (*n* = 2) combination, for a total of eight regressions.

### 2.8 Data analysis: partial barriers and interannual variation in distribution of genotypes

Next, we explored the influence of partial barriers on the distribution of migratory genotype fish in Elder Creek, including across dry and wet years. For this analysis, we focused on migratory alleles of juvenile (young of year) fish, because their sample location probably reflects where their parents bred and where they hatched (Hudy, Coombs, Nislow, & Letcher, 2010). Hence juvenile location is a proxy for the passage ability of steelhead adults the previous winter. We classified individuals as young‐of‐year fish, hereafter referred to as “juveniles,” if they were <85 mm in fork length (Kelson, Power, Finlay, & Carlson,^2020^). We tested the interannual variability in permeability of partial barriers, and predicted that they would be less permeable to upriver migrating steelhead in dry years. As a result, in dry years, we predicted that we would find higher densities of migratory alleles downstream of each barrier.

Within Elder Creek, we explored the interannual variation in migratory allele frequency at the three partial barriers (Elder waterfall and two tributary confluences). To test the effect of tributary confluences, we compared the number of migratory alleles per study pool in the tributary versus the reach of Elder Creek above the waterfall (i.e., excluding pools downstream of the large barrier). For each potential partial barrier, we conducted a generalized linear model (Poisson distribution) with the number of migratory alleles per sample pool as the response variable and with sample location (downstream or upstream of the partial barrier), year and surface area (m^2^) of the pool as predictor variables. We tested for an interaction between year and sample location (downstream vs. upstream of the barrier). A significant interaction indicates that the difference in migratory alleles per pool downstream versus upstream of each feature depends on the year. We then compared the full model (including year × location interaction) with the reduced model (no interaction) using an *F* test. When the full model (with interaction) explained significantly more variation than the reduced model, indicating that the effect of the partial barrier differed among years, we ran the model for each year separately.

Within Fox Creek, the major putative barrier is located at the creek mouth, so we tested for interannual variation in the density of migratory alleles (number per pool) for the entire creek. Here, we conducted a generalized linear model (Poisson distribution) with number of migratory alleles per pool as the response variable, and including sample year and pool surface area (m^2^) as predictor variables.

## 3 RESULTS

### 3.1 Interannual variation in stream flow and barrier passage

**Figure 2:**
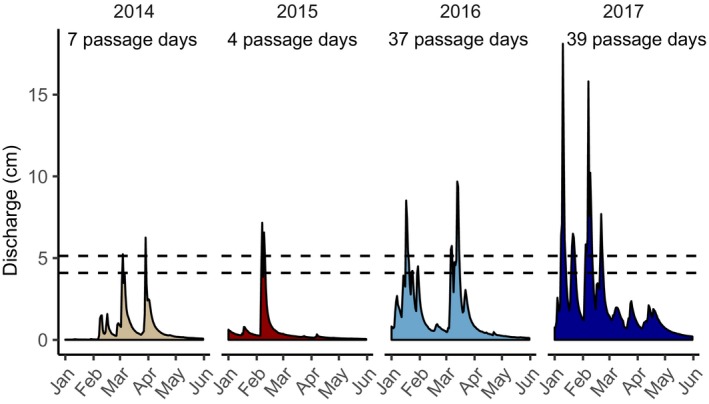
Stream flow patterns in Elder Creek from 2014 to 2017 during the steelhead breeding window, which included two dry years (2014 and 2015) and two wet years (2016 and 2017). Dotted lines indicate the flow window when the waterfall in Elder Creek is estimated to be passable to adult steelhead. Passage days are when the daily mean stream flow falls within the passage flow window [Colour figure can be viewed at http://wileyonlinelibrary.com]

Our study encompassed two dry years (2014, 2015) and two wet years (2016, 2017), based on the average DSCI in the South Fork Eel River watershed during the steelhead breeding window (January–May, DSCI score of 392 and 324 in 2014 and 2015; 120 and 0 in 2016 and 2017). Beyond differences in total precipitation, there were differences in the magnitude and timing of high‐flow events during the adult steelhead breeding season. In 2014, stream flows were elevated in March and April, while in 2015 the only major flow event occurred in February (Figure 2). Both 2016 and 2017 were characterized by higher stream flows overall during the adult steelhead spawning season, with the highest flows in 2016 in January and March and several high‐flow events distributed throughout the breeding season in 2017 (Figure 2). Using the estimated flow passage window based on results of Trush (1989), the waterfall on Elder Creek was passable for 7 days in 2014, 4 days in 2015, 37 days in 2016 and 39 days in 2017.

### 3.2 Genetic diversity at neutral versus migratory loci

We found that *Omy05* SNPs were characterized by lower heterozygosity than expected (*H*_0_ = 0.43, *H*_E_ = 0.46, *p* < .01 in a paired *t* test), and this was the only chromosome where this pattern was observed (Figure S1), which is consistent with positive assortative mating with respect to this chromosome (i.e., individuals are more likely to mate if they have similar *Omy05* genotypes and migration phenotypes; Miller et al., 2012; Pearse et al., 2014; Pearse et al., 2019). *F*_ST_ comparisons using putatively neutral markers (i.e., with *Omy05* excluded) between the streams (within regions of Elder Creek) and Fox Creek were all < 0.02 and not statistically significant. Together these results suggest little genetic divergence among *Oncorhynchus mykiss* captured from neighbouring locations.

We found several SNPs on both *Omy02* and *Omy06* that had a high frequency of heterozygous genotype calls (Figure S1), both of which appear to have retained residual tetrasomic inheritance (Sutherland et al., 2016). These SNPs had little to no effect on our PCA (Figure S2) because they have little to no variation among individuals (i.e., they are called as heterozygous in nearly all individuals). In more detail, the ancestor to all salmonids underwent a whole genome duplication ~65 million years ago (Sutherland et al., 2016). Since this tetraploidization event, the process of rediploidization has been occurring and has produced to two categories of paralogous genomic regions in contemporary salmonids: (a) paralogous regions that have diverged substantially because there is no longer recombination between the paralogues (i.e., regions that have rediploidized), and (b) paralogous regions that have retained tetrasomic inheritance (i.e., regions that still have recombination between paralogues), which prevents substantial divergence between the paralogues. Although most of the genome has rediploidized, tetrasomic inheritance still occurs (or has at least occurred in the recent evolutionary past) on the distal end of a subset of chromosome arms (Allendorf et al., 2015; Sutherland et al., 2016). The alignment of sequence reads from regions that retain tetrasomic inheritance can result in paralogous sequence variants, which manifest as SNPs with a high frequency of heterozygous genotype calls (e.g., approaching 100%) (Waples, Seeb, & Seeb, 2017).

For the PCA, which excluded SNPs on *Omy05*, there was no strong clustering, with 80% of the samples falling in a centre cluster and no strong clustering based on sample location, including samples collected from the South Fork Eel River (Figure S2). Additionally, there was no clustering by year or data quality (number of SNPs missing data per individual) (Figure S2). A subset of resident and migratory genotypes diverged from the centre circle, but again, the majority (80%) of samples fell within the centre cluster (Figure S2). Loadings for the PCs were distributed across many SNPs throughout the genome (Figure S2). Together these results suggest that adaptive variation has a strong pattern of spatial distribution while overall genetic structure is very low.

### 3.3 Spatial patterns in proportion of migratory alleles

**Figure 3:**
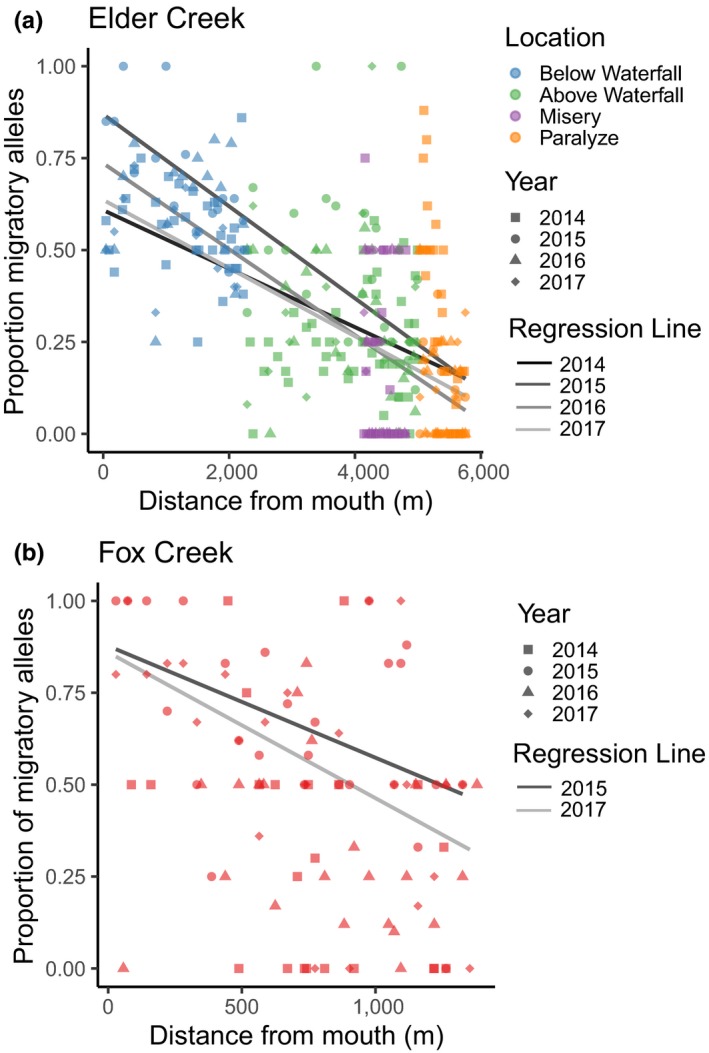
(a) The proportion of migratory alleles per pool decreases with distance upstream in Elder Creek in 2014–2017 (significant in all years). (b) The proportion of migratory alleles per pool shows a weak but negative relationship with distance upstream in Fox Creek (significant in 2015 and 2017) [Colour figure can be viewed at http://wileyonlinelibrary.com]

To explore the overall spatial patterns in migration, we explored the longitudinal distribution of migratory *O. mykiss* for all age classes. Within Elder Creek, migratory allele frequency decreased with upstream distance. There was a strong, linear relationship between the proportion of migratory alleles per pool and the distance upstream in each year (Figure 3a, 2014: *r* ^2^ = .27, *z* = −6.3, *p* < .001, 2015: *r* ^2^ = 0.50, *z* = −6.9, *p* < .001, 2016: *r* ^2^ = 0.52, *z* = −7.5, *p* < .001, 2017: *r* ^2^ = 0.39, *z* = −5.6, *p* < .001). Within Fox Creek, there was a weak, but significant, linear relationship between the proportion of migratory alleles per pool and upstream distance in 2015 and 2017 (Figure 3b, 2015: *r* ^2^ = 0.21, *z* = −2.4, *p* < .05, 2017: *r* ^2^ = 0.26, *z* = −2.9, *p* < .01), and no relationship in 2014 or 2016 (2014: *r* ^2^ = 0.13, *z* = −1.6; *p* = .12, 2016: *r* ^2^ = 0.01, *z* = −0.43, *p* = .67).

### 3.4 Partial barriers and interannual variation in spatial distribution of genotypes

**Figure 4:**
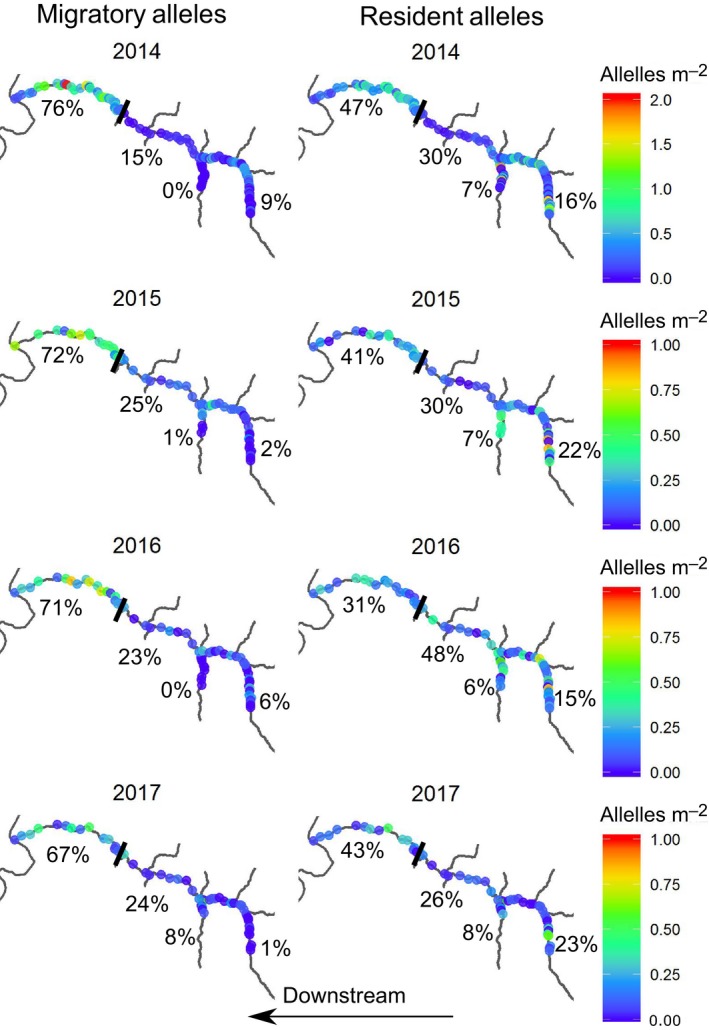
Distribution of migratory alleles per m^2^ for juvenile fish in Elder Creek across 4 years of sampling. Each dot represents a sample pool. The solid black line is the location of the waterfall. Percentages represent the proportion of all migratory alleles for juveniles in the creek that are located in each stream region (all four regions together sum to 100%, i.e., the total migratory alleles for juvenile fish found in Elder Creek in that year). Note the different scale in 2014 than other years due to higher density of migratory genotype fish in this year [Colour figure can be viewed at http://wileyonlinelibrary.com]

**Figure 5:**
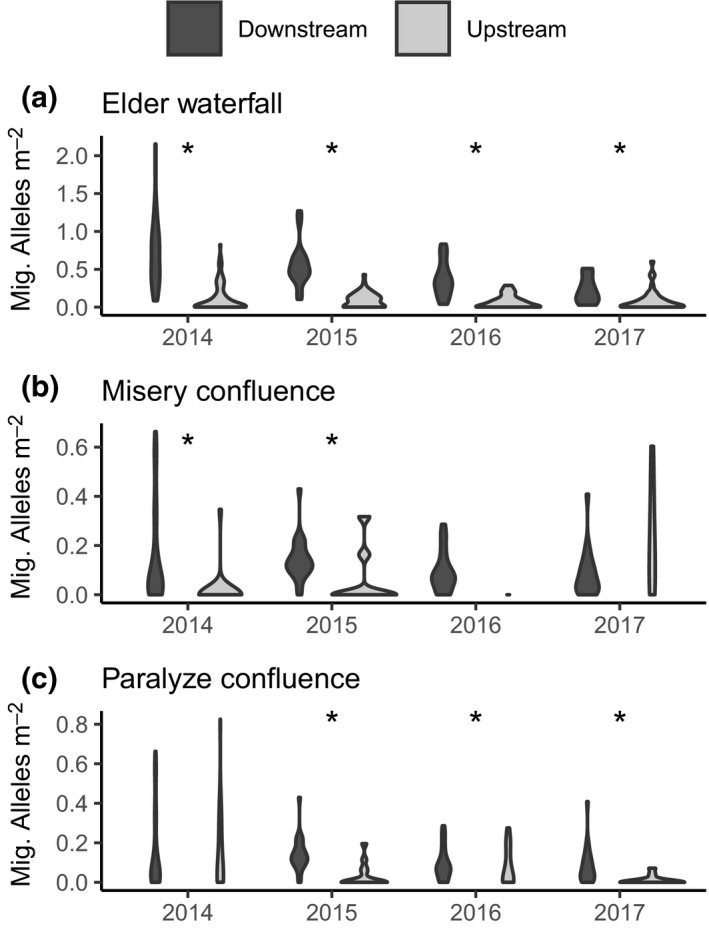
Violin plots showing that the density of migratory alleles was always lower upstream of partial barriers, but the difference in density between sample pools upstream and downstream of each barrier varied among years. (a) At the Elder Creek waterfall, the difference was greatest in 2014 and 2015, but significant in all years. (b) At the Misery Creek confluence this difference was not significant in wet years, 2016 and 2017. (c) At the Paralyze Creek confluence the difference was lowest and not significant in 2014. Upstream–downstream comparisons that were significant in a generalized linear model are noted with asterisks

We found that partial barriers influenced the distribution of genotypes. In particular, within Elder Creek, the density of juvenile migratory alleles was higher downstream than upstream for each of the three partial barriers: the waterfall, Misery confluence and Paralyze confluence (Figures 4 and 5). The waterfall had the strongest effect in reducing the number of upstream migratory alleles in each year, and as a result the majority of migratory alleles in the watershed were found downstream of this barrier (67%–76%, Figure 4). In contrast, resident alleles were distributed more evenly throughout the watershed, with 31%–47% being found downstream of the barrier (Figure 4). While these larger patterns were consistent among years, we found some among‐year differences in the distribution of migratory alleles (Figure 5). Specifically, in our generalized linear models, the effect of each partial barrier on the density of migratory alleles upstream of the feature varied among years (the location × year interaction was significant, and favoured in the *F* test). Consequently, we conducted generalized linear modelling of migratory alleles found upstream versus downstream of each barrier separately for each year. For the waterfall in Elder Creek, the difference in the number of migratory alleles per study pool between the downstream and upstream regions was highest in 2014 and 2015 (Figure 5), and this difference was significant in all years (Table S1). For Misery Creek, there were more migratory alleles in the upstream creek than in regions downstream of the confluence in the dry years of 2014 and 2015. Correspondingly, the effect of sample location (upstream vs. downstream of the confluence) was significant in 2014 and 2015, but not in 2016 or 2017 (Table S1). Within Paralyze Creek, the density of migratory alleles in the creek was not different from the density downstream of confluence in 2014, the driest year (Figure 5; Table S1). Indeed, in this year, migratory alleles were more common in Paralyze than in any other year. In all other years in Paralyze, there were fewer migratory alleles per study pool in the upstream creek than downstream of the confluence (Figure 5).

**Figure 6:**
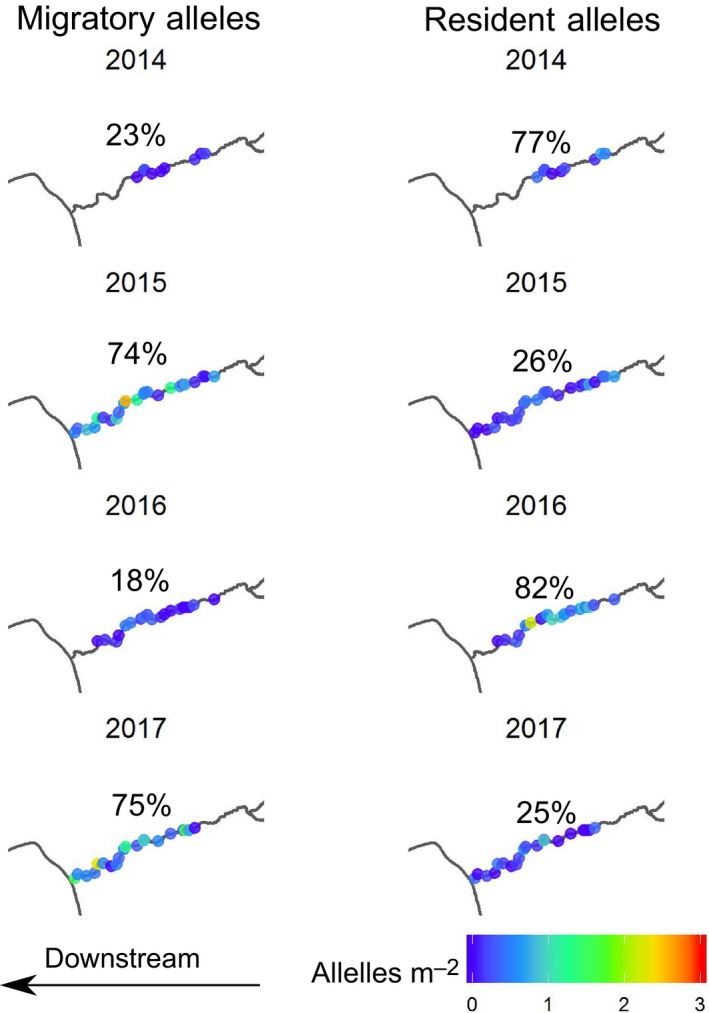
Distribution of migratory versus resident alleles for juvenile fish in Fox Creek across 4 years of sampling, 2014–2017. Percentages refer to the allele (migratory or resident) frequency for the entire stream for that year for fish of all age classes [Colour figure can be viewed at http://wileyonlinelibrary.com]

For Fox Creek, the strongest pattern in migratory alleles was across years, rather than spatially within the watershed (Figure 6). We found strong interannual variation in the number of migratory alleles per pool for juvenile fish, with year explaining 35% of the deviance (318.5 out of a total 909.6) in an ANOVA. There was a strong increase in migratory alleles per pool in the years of 2015 and 2017, in comparison to 2014 (2015 estimate ± *SE* = 4.7 ± 1.1, *t* = 4.1, *p* < .01; 2017 estimate ± *SE* = 4.6 ± 1.2, *t* = 4.0, *p* < .01, see also Figure 6). There was no increase in the number of migratory alleles per pool in 2016 compared to 2014 (estimate ± *SE* = 0.6 ± 1.2, *t* = 0.5, *p *= .62). The overall migratory allele frequency varied among years, and was low (30% and 30%) in 2014 and 2016, and high (68% and 60%) in 2015 and 2017. When focusing on the migratory allele frequency of juvenile fish, among‐year differences were even more extreme (23% and 18% in 2014 and 2016; 74% and 75% in 2015 and 2017, Figure 6).

## 4 DISCUSSION

Our results highlight that among‐year variation in stream flows can influence landscape permeability. In particular, we found that small partial barriers concentrated migratory genotypes in downstream reaches. There was interannual variation in the effect of these barriers; partial barriers were generally less permeable during dry years. This pattern was especially true for the smaller population, Fox Creek, where the migratory allele frequency was reduced by over 50% in two years, which were years when high stream flow events did not align with peak migration and breeding timing. This result highlights the ability of migratory animals to move into habitat areas when they are accessible and the importance of windows of access, which may vary across years. These temporarily available habitats may be zones where there is frequent, rapid change in allele frequencies that are associated with whether migratory animals have access. Our study provides an example of how monitoring changes in the spatial distribution of adaptive genetic variation in migratory animals can illuminate temporal variability in landscape permeability, and will be useful in predicting how genetic variation will change alongside climate and landscapes.

### 4.1 Distance and permeable barriers influence distribution of migratory genotypes

Theory suggests that migratory tendencies should decrease when costs of migration are high (Alerstam, Hedenström, & Åkesson, 2003). In salmonid fishes, cost of migration is often approximated as distance travelled or elevation difference between the ocean rearing and freshwater breeding sites (Hendry, Bohlin, Jonsson, & Berg, 2003; Jonsson & Jonsson, 1993). Consistent with theoretical predictions, migratory trout tend to be distributed further downstream while resident trout are concentrated upstream, a pattern that has been observed in large watersheds in *Oncorhynchus mykiss* (Berejikian et al., 2013; McMillan, Katz, & Pess, 2007; Narum et al., 2008) and in brown trout (*Salmo trutta)* (Bohlin, Pettersson, & Degerman, 2001). Here, we found that stream distance reduces the proportion of migratory alleles even within a small watershed with a long zone of co‐occurrence, that is within 6 km of stream in Elder Creek, and to a lesser extent within 2 km of stream in Fox Creek.

Partial barriers also influenced the spatial distribution of migratory alleles in juvenile fish. Within Elder Creek, the reduction in migratory allele frequencies was greatest at the largest partial barrier, a waterfall located relatively low within the watershed (~2 km from the mouth). The majority of migratory alleles (67%–72%) in the system were found in the reach of stream below this partial barrier. While other studies have documented that complete barriers select against anadromy, leading to divergence in *O. mykiss* populations distributed above and below barriers (Leitwein, Garza, & Pearse, 2017; Pearse et al., 2009), our study provides an example of the lesser‐studied effects of small, partial barriers on the distribution of migratory genotypes in *O. mykiss.* Small barriers are common across the landscape (Januchowski‐Hartley et al., 2013), and include natural features, such as tributary confluences or waterfalls like those studied here, but also include artificial landscape features, such as road crossings (Benton, Ensign, & Freeman, 2008) and weirs, or small diversion dams (Newton, Dodd, Barry, Boylan, & Adams, 2018; Weigel, Connolly, & Powell, 2013). Apgar et al. (2017) estimated many small barriers can have a similar effect as a single larger barrier in reducing the migratory allele frequency in *O. mykiss*. While small, partial barriers often get less attention in studies of landscape fragmentation, and they may be just as important in terms of their cumulative impact as single large barriers in determining how traits, populations and communities are distributed across the landscape.

Here, we demonstrate that landscape permeability, including distance upstream and partial barriers, determines the potential for migratory animals to access certain habitats and thus the spatial distribution of migratory genotypes. Once a habitat is occupied by both resident and migratory individuals, the individual decision to migrate is often considered a threshold trait (Pulido, 2011). Furthermore, partial migration can be considered an evolutionary stable strategy such that migration is condition‐dependent (Lundberg, 1987), and strongly influenced by the density of conspecifics (De Leenheer, Mohapatra, Ohms, Lytle, & Cushing, 2017). Together, these processes may create feedback loops, such that habitats that can be accessed by migratory animals tend to have higher densities of juveniles (Bohlin et al., 2001; Nilsson, Lindström, Jonzén, Nilsson, & Karlsson, 2006), which can then encourage migration (Kaitala, Kaitala, & Lundberg, 1993; Taylor & Norris, 2007). Thus, overall, the landscape features that are en‐route for migratory animals play a role shaping selection at the population level (Micheletti et al., 2018) and perhaps, ultimately, the production of migratory individuals.

### 4.2 Interannual variation in migratory allele frequencies and landscape permeability

Beyond the winnowing influence of partial barriers, we documented interannual variation in their permeability. This pattern was most pronounced in Fox Creek, where in two years (2014 and 2016), the migratory allele frequency was reduced by more than 50% due to the apparent inability of migratory fish to ascend the barrier at the mouth of the creek. This pattern was more extreme in 2014, when only 1.2% (one out of 82 age‐0 fish sampled) of juveniles had migratory genotypes. There were no high‐flow events in February of 2014 or 2016, which is the peak breeding season for steelhead trout in the Eel River watershed (Brown, 1990; Trush, 1989). In Elder Creek, the timing of the high flows seemed to be less important in terms of access to the creek, and instead the amount of high flows influenced the distribution of migratory alleles within the creek. We found that migratory alleles were more dense downstream of the waterfall in the two driest years (2014 and 2015). Because the mouth is always navigable, Elder Creek may attract steelhead trout in years of low flow during the upriver migration. In support of this idea, the densities of migratory alleles below the waterfall in Elder Creek where highest in the dry years, 2014 and 2015, which contributed to the greater difference above and below the waterfall in these years compared to the wet years. These results emphasize that the influence of partial barriers on distributions and gene flow is dynamic, with passage of migratory individuals depending on the timing and magnitude of high‐flow conditions, in addition to passage conditions at other locations throughout the watershed. Additionally, the influence of partial barriers may depend on the number of adults migrating up‐river (run size) in the watershed in a given year. Future studies could investigate the relative impacts of run size compared to the permeability of partial barriers on genotype frequencies in watersheds where both data sets are available.

The interannual variation in migratory allele frequencies that we found suggests that adults migrating up‐river expand their range upstream of partial barriers when conditions allow. Similar results of upstream range expansion have been observed following dam removal (Kiffney et al., 2009; McMillan et al., 2015; Weigel, Connolly, Martens, & Powell, 2013), when upstream migrating fish have recolonized former habitat following barrier removal. Like many diadromous fishes, the distribution of migratory *O. mykiss* has been reduced by dams, and restoring migration is a major goal where this life history has been lost (Limburg & Waldman, 2009; Quiñones et al., 2014). Our results suggest that upstream range expansion of the migratory life history is possible when barrier permeability is increased.

Temporal variability in movement following changes in landscape permeability has been demonstrated in other systems. For example, in the Canadian Rockies, seasonal variation in the number of vehicles per day on major highways affects large mammal crossings (Alexander, Waters, & Paquet, 2005). Another example comes from temporary rivers, where the cessation of flow and stream drying creates a movement corridor for terrestrial animals and insects (Sánchez Montoya, Moleón, Sánchez‐Zapata, & Escoriza, 2017; Steward, Schiller, Tockner, Marshall, & Bunn, 2012). However, the influence of interannual variation in environmental conditions on barrier permeability has rarely been explored in the context of genetic diversity and gene flow. Some exceptions include studies that compare genetic diversity in historical versus contemporary samples (Heath, Busch, Kelly, & Atagi, 2002; Martínez‐Cruz, Godoy, & Negro, 2007) or long‐term gene flow estimates (*F*_ST_ values) coupled with contemporary movement data (Epps, Wasser, Keim, Mutayoba, & Brashares, 2013). Studies that use individual‐based, spatially and temporally explicit sampling or modelling (Landguth, Cushman, Murphy, & Luikart, 2010; Landguth, Muhlfeld, & Luikart, 2011) may be powerful approaches for disentangling the influence of among‐year variation in the environment and landscape features, as well as their interaction, on patterns of gene flow and genetic diversity, and especially adaptive genetic diversity that is under strong selection.

### 4.3 Migratory‐linked loci shape genetic structure in partially migratory populations

We found no genetic divergence between streams or above versus below partial barriers within a stream when using putatively neutral loci (*F*_ST_ < 0.02). These results suggest that resident and migratory individuals are interbreeding and maintaining gene flow over long timescales. Additionally, the lack of divergence between the two streams, Fox and Elder Creeks, suggests that steelhead are not necessarily returning to their natal creek to breed in the South Fork Eel River watershed. This may be due to the presence of intermittently permeable barriers, which could force fish to breed outside of their natal stream in years when they are inaccessible. Apgar et al. (2017) also found that migration‐linked loci frequencies showed greater divergence than neutral loci in *O. mykiss*, and Van Doornik, Berejikian, and Campbell (2013) reported high rates of gene flow between sympatric anadromous and resident *O. mykiss*. The result also aligns with evidence that anadromous and resident *O. mykiss* within a watershed are more closely related to each other than to fish with the same life history in neighbouring watersheds (Clemento, Anderson, Boughton, Girman, & Garza, 2009; Leitwein et al., 2017; Olsen, Wuttig, Fleming, Kretschmer, & Wenburg, 2006).

Our result that a migration‐linked trait does not lead to neutral genetic divergence parallels results from diverse systems, from birds to fish (Bensch, Åkesson, & Irwin, 2002; O’Malley, Camara, & Banks, 2007; O’Malley et al., 2013). Migration is often linked to a narrow region in the genome (Liedvogel, Åkesson, & Bensch, 2011), and is often associated with dramatic phenotypic changes in migrating individuals. Studying temporal and spatial changes in migration‐linked genetic variation may indicate which taxa are most likely to continue expressing migration in the face of changes to landscape permeability. Migratory animals that are able to alter their routes to avoid detours or make use of stop‐over habitats such as ungulates (Sawyer et al., 2013), or alter their timing of migration such as many birds (reviewed in Gill et al., 2014; Gordo, 2007), may be more likely to continue to express migration in fragmented landscapes when environmental conditions render particular partial barriers impassible. More generally, understanding how the spatial distribution of migration‐linked loci varies through time in many taxa could reveal which landscape features and environmental conditions select against migration, which is on the decline globally (Wilcove & Wikelski, 2008).

## 5 CONCLUSIONS

Overall, our results emphasize the dynamic nature of partial barriers on the distributions and genetic diversity of migratory animals. In river systems, permeability of partial barriers is mediated by river flows; in general, partial barriers are more permeable when high flows coincide with the timing of migrations. More generally, the spatial distribution of resident and migratory individuals in partially migratory populations is likely to be dynamic and influenced by landscape features and environmental variability. Using genetic tools to explore temporal shifts in allele frequencies at loci associated with migratory traits may help to reveal temporal variation in landscape permeability and the consequences for the distribution of migratory animals.

## AUTHOR CONTRIBUTIONS

S.J.K. led study design and sample collection, conducted laboratory work and statistical analyses, and wrote the manuscript. T.Q.T conducted bioinformatics. S.M.O. conducted laboratory work and analyses. M.R.M. contributed to study design and advised bioinformatics. S.M.C. contributed to study design, assisted with field sample collection, and advised analyses. All authors contributed to writing and editing the manuscript.

## Data Availability

Raw sequence data are available at NCBI, SRA accession: PRJNA599015. Other data are available on Dryad: Kelson et al. (2020), Temporal dynamics of migration‐linked genetic variation are driven by streamflows and riverscape permeability, v2, UC Berkeley, Dataset, https://doi.org/10.6078/D1DM6G. This dryad deposit includes: IBS matrix (single‐read SNPs) for SNPs missing a maximum of 20% of data and for SNPs missing a maximum of 50% of data, data frame of called genotypes for SNPs missing a maximum of 20% of data, resident and migratory genotypes on *Omy05* for all fish, and habitat data used to calculate density during electrofishing surveys.
