# Supplementary material for: Temporal dynamics of migration‐linked genetic variation are driven by streamflows and riverscape permeability
Source: Mol Ecol. 2020 Feb 17;29(5):870–85. doi: 10.1111/mec.15367 (PMC7078995; doi:10.1111/mec.15367)
Supplement: Supplementary file 1 [file MEC-29-870-s001.pdf]

Supplemental Information for:

Temporal dynamics of migration-linked genetic variation are driven by streamflows and riverscape permeability.

Kelson, Suzanne J., Miller, Michael R., Thompson, Tasha Q., O'Rourke, Sean M., Carlson, Stephanie M.

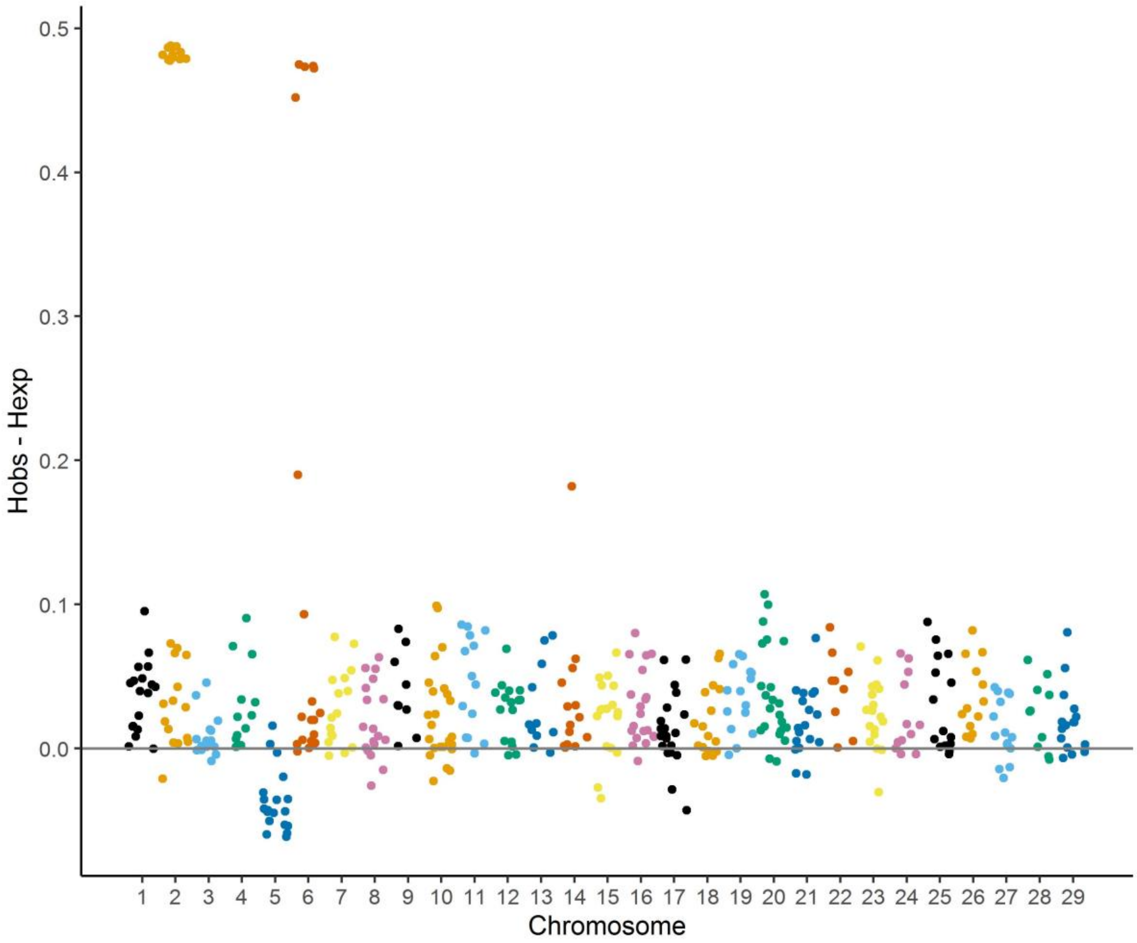


**Figure S1.** Loci on *Omy05* were less heterozygous than expected, and this pattern was more extreme and consistent for loci on *Omy05* than for loci that were on other chromosomes. Loci on Omy02 and Omy06 were more heterozygous than expected, likely due to the residual tetrasomic inheritance found on these chromosomes (see Materials and Methods; Waples et al. 2016; Sutherland et al. 2016).

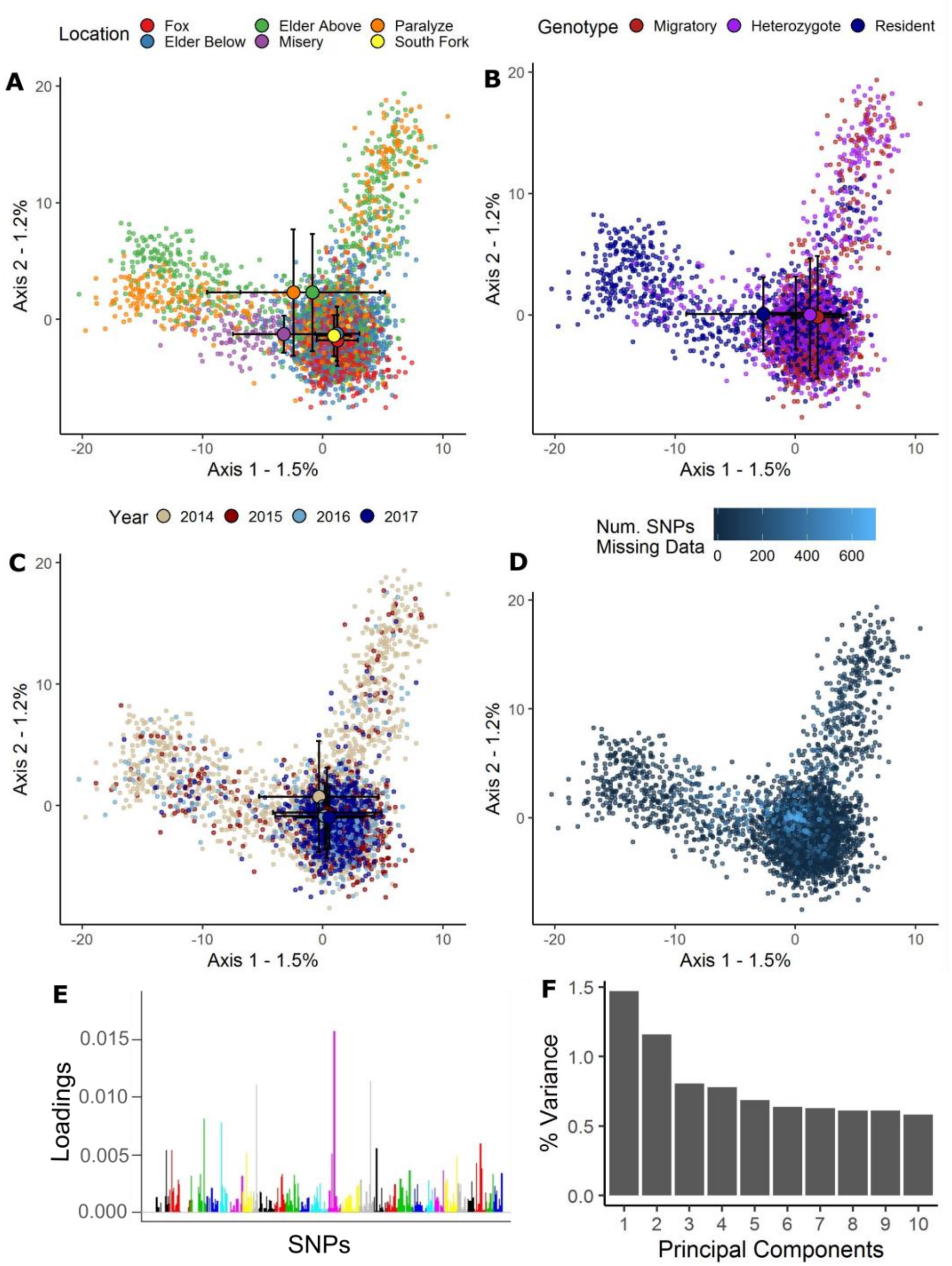


**Figure S2.** A PCA excluding SNPs on *Omy05* (n = 692 SNPs) demonstrates very little genetic structure among Fox Creek and regions within Elder Creek (above and below the waterfall, and Misery and Paralyze tributaries), but a strong influence of the life history-associated genotypes. The PCA is colored by A) sample location B) life history genotype C) sample year and D) number of SNPs that are missing data. Categorical variables are summarized by means and standard deviation (plots A-C). E) A loading plot averaging over the all principal components demonstrate that variance is distributed evenly across chromosomes. Colors represent chromosomes. Plot F) and shows the percent variance explained by each principal component.

**Table S1.** Parameter coefficients and p-values for generalized linear models testing the influence of each barrier on the density of migratory alleles in each sample year. Models were run for each barrier in Elder Creek (waterfall, two tributary confluences), and for each year. Parameters that are italicized are not significant at the $P<0.05$ level. Years that are in bold are years where there is no significant difference in migratory alleles downstream vs. upstream of the barrier.

| **Barrier** | **Year** | **Parameter** | **Estimate (Std. Error)** |
|---|---|---|---|
| Elder Waterfall | 2014 | Intercept | 2.65 (0.06) |
| | | Surface Area | 0.01 (0.00) |
| | | Upstream | -2.14 (0.08) |
| | 2015 | Intercept | 1.88 (0.10) |
| | | Surface Area | 0.02 (0.00) |
| | | Upstream | -1.70 (0.12) |
| | 2016 | Intercept | 1.36 (0.18) |
| | | Surface Area | 0.03 (0.01) |
| | | Upstream | -1.75 (0.15) |
| | 2017 | Intercept | 1.94 (0.21) |
| | | *Surface Area* | 0 .00 (0.00) |
| | | Upstream | -1.78 (0.19) |
| Misery Confluence | 2014 | Intercept | 1.18 (0.13) |
| | | *Surface Area* | 0.00 (0.00) |
| | | Upstream | -4.30 (1.01) |
| | 2015 | Intercept | 0.84 (0.19) |
| | | Surface Area | 0.02 (0.00) |
| | | Upstream | -1.94 (0.60) |
| | **2016** | Intercept | 0.62 (0.37) |
| | | *Surface Area* | 0.01 (0.01) |
| | | *Upstream* | -18.98 (1807.83) |
| | **2017** | Intercept | 0.73 (0.32) |
| | | *Surface Area* | -0.01 (0.01) |
| | | *Upstream* | -0.21 (0.40) |
| Paralyze Confluence | **2014** | Intercept | 1.01 (0.12) |
| | | *Surface Area* | 0.00 (0.00) |
| | | *Upstream* | -0.15 (0.15) |
| | 2015 | Intercept | 0.82 (0.19) |
| | | Surface Area | 0.02 (0.00) |
| | | Upstream | -1.65 (0.38) |
| | 2016 | *Intercept* | 0.57 (0.35) |
| | | *Surface Area* | 0.01 (0.01) |
| | | Upstream | -0.87 (0.34) |
| | 2017 | Intercept | 0.74 (0.32) |
| | | *Surface Area* | -0.01 (0.01) |
| | | Upstream | -2.33 (0.73) |
